# Improving gene transfer in *Clostridium pasteurianum* through the isolation of rare hypertransformable variants

**DOI:** 10.1016/j.anaerobe.2017.09.001

**Published:** 2017-12

**Authors:** Alexander Grosse-Honebrink, Katrin M. Schwarz, Hengzheng Wang, Nigel P. Minton, Ying Zhang

**Affiliations:** Clostridia Research Group, BBSRC/EPSRC Synthetic Biology Research Centre (SBRC), School of Life Sciences, Centre for Biomolecular Sciences, University of Nottingham, University Park, Nottingham, NG7 2RD, United Kingdom

**Keywords:** *Clostridium pasteurianum*, DNA transfer/transformation efficiency, Structural maintenance of chromosomes (SMC)

## Abstract

Effective microbial metabolic engineering is reliant on efficient gene transfer. Here we present a simple screening strategy that may be deployed to isolate rare, hypertransformable variants. The procedure was used to increase the frequency of transformation of the solvent producing organism *Clostridium pasteurianum* by three to four orders of magnitude.

## Highlights

• A procedure to isolate *C. pasteurianum* mutants capable of high efficient DNA transfer.
• Increase DNA transfer efficiency by three to four orders of magnitude.
• Structural maintenance of chromosome proteins of *C. pasteurianum* are involved in DNA transfer.

Metabolic engineering of microbial chassis is reliant on effective methods of DNA transfer. One commonly used route to achieve the necessary high frequencies is to implement rational strategies designed to overcome the barrier imposed by host restriction-modification (R/M) systems [1]. Recently, we adopted a different approach that was reliant on the isolation of hypertransformable variants within the general population of the solvent producing strain *Clostridium pasteurianum* [2]. In our initial work the frequency of DNA transfer was such that only a handful of antibiotic resistant transformants were obtained following electroporation of competent cells of the *C. pasteurianum* strain DSM 525 with several *E.coli/Clostridium* shuttle vectors. We hypothesised that the few transformants that were obtained were most likely a result of the presence of rare mutant variants within the culture that were highly competent for DNA transfer. This notion was tested by curing one of the rare transformants of its acquired plasmid, and then testing the frequency of DNA transfer in the isolated plasmid-free strain obtained. The plasmid-cured strain, designated *C. pasteurianum* DSM 525-H1 (CRG4111), was found to transform at frequencies of up to 10^5^ transformants per μg DNA [2]. High throughput genome sequencing revealed that frame-shift mutations had arisen in two independent genes. The one (CLPA_c13710, *ablB*) encoded a predicted β-lysine N6-acetyltransferase while the other (CLPA_c33080, *resE9*) encoded a histidine kinase likely to be involved in quorum sensing. Neither gene appeared to encode a protein which played any direct role in R/M.

To further explore our hypothesis, here we took a more methodical approach to screening DSM 525 for the presence of hypertransformable variants. In essence, a total of six independent batches of competent cells were prepared from a glycerol stock of *C. pasteurianum* DSM 525 and transformed with 5 μg of *in vivo* M.*Bep*I-methylated pMTL85151 [3] plasmid DNA (see Supplementary data for details). A single thiamphenicol (Tm) resistant (^R^) transformant colony from each electroporation was used to inoculate 5 ml of antibiotic-free 2xYTG broth (16 g/l tryptone, 10 g/l yeast extract, 4 g/l NaCl, 5 g/l glucose, pH 6.5). Following growth, cells were serially diluted onto RCM (Reinforced Clostridial Medium) agar without Tm and the subsequently developed colonies were patch-plated onto RCM and RCM + Tm agar plates. The pIM13 replicon of plasmid pMTL85151 is very unstable in *C. pasteurianum* DSM 525 [2], hence on average over 97.2 ± 4.1% of the clones tested had become Tm sensitive (^S^), indicative of plasmid loss. A randomly selected Tm^S^ clone from each of the six cell lines was chosen and, through colony PCR and pMTL8515-specific primers (Table S1), shown to have lost the plasmid. Competent cells prepared from these six plasmid-cured cell lines were used in subsequent transformation experiments with M.*Bep*I methylated pMTL85151 plasmid DNA. In two instances (cell lines 525-H3 and 525-H4) dramatically increased transformation frequencies were obtained, some 2-3 orders of magnitude higher than obtained with wildtype DSM 525 competent cells and comparable to those seen with the previously isolated [2] hypertransformable strain 525-H1 (Table 1).

**Table 1:** Transformation efficiencies of six independent screenings before and after plasmid curing. Experiments 3 and 4 showed significant increase of DNA transfer efficiency.

Sample	Transformation frequency before curing plasmid [cfu/μg DNA]	Transformation frequency after curing plasmid [cfu/μg DNA]
1	0.8 × 10^2^	5.2 × 10^2^
2	1.3 × 10^2^	1.8 × 10^2^
3	1.3 × 10^2^	1.8 × 10^5^
4	1.6 × 10^2^	0.7 × 10^5^
5	1.6 × 10^2^	0
6	1.7 × 10^2^	2.0 × 10^1^
wild type 525	n.a.	1.2 × 10^2^
525-H1	n.a.	3.4 × 10^4^

To determine the molecular basis for improved transformation rates, paired-end chromosomal DNA libraries of 525-H3 and 525-H4 were prepared and subjected to Illumina sequencing on a MiSeq sequencer and the reads obtained mapped against the published *C. pasteurianum* DSM 525 genome [4] (GenBank Accession Number CP009268) using CLC Genomics Workbench 8.0.2 (Qiagen, DK). In both strains a single identical SNP was found at reference nucleotide position 3284805, in which an adenine was replaced with a cytosine nucleotide. This substitution resulted in replacement of the phenylalanine residue at position 1102 of the encoded protein, CLPA_c30550, with a valine. Sanger sequencing (Source Bioscience Nottingham, UK) was used to confirm the presence of the A > C substitution at position 3284805 in both 525-H3 and 525-H4. This SNP was additionally shown to be absent in the previously isolated [2] hypertransformable variant 525-H1. Similarly, the two mutations present in 525-H1 [2] were shown to be absent in 525-H3 and 525-H4.

To definitively establish that the identified SNP in strains 525-H3 and 525-H4 was responsible for the observed increase in transformation frequency we elected to restore the mutation responsible back to the wildtype. This was achieved by allelic exchange, essentially as previously described [2] but in this case using the *codA* gene as the counter selection marker [5] as opposed to a *pyrE* gene [2]. The necessary vector was made by replacing the *Fse*I and *Pme*I DNA fragment of pMTL-KS15 that encompasses *catP-pyrE* [2] with an equivalent module specifying *codA-catP* (see Supplementary data for details). The new plasmid was designated pMTL-AGH15 (Fig. S1). Into this plasmid was cloned a 1127 bp *Sbf*I-*Nhe*I fragment encompassing the wild type CLPA_c30550 allele, yielding the SNP repair plasmid pMTL-AGH15-c30550. The cloned fragment was generated by PCR using DSM 525 chromosomal DNA as the template and the primers Segr_LHA_SbfI and Segr_RHA_NheI_R (see Supplementary data for details). Thereafter, the plasmid pMTL-AGH15-c30550 was *in vivo* methylated, electrotransformed into 525-H3 and putative single crossover integrant tranformants selected as faster growing colonies on RCM + Tm agar. A total of 24 such colonies were streaked to purity on RCM + Tm agar, and then restreaked a further two times on antibiotic-free CBM (Clostridial Basal Medium [6]) containing 500 μg/ml fluorouracil (FC) (Sigma Aldrich, UK) to counter-select for FC^R^ cells in which the plasmid had excised and been lost. Fourteen randomly selected FC^R^ clones were shown to be Tm^S^ (did not grow on RCM + Tm), indicative of plasmid loss. To establish whether the SNP in CLPA_c30550 had been corrected to wild type, PCR primers Segr_flank_F and Segr_flank_R were used to amplify the region of CLPA_c30550 encompassing the SNP and subjected to Sanger sequencing. In total, 7 of the clones tested carried the original SNP, whereas in the remainder it had been restored.

**Fig. 1:**
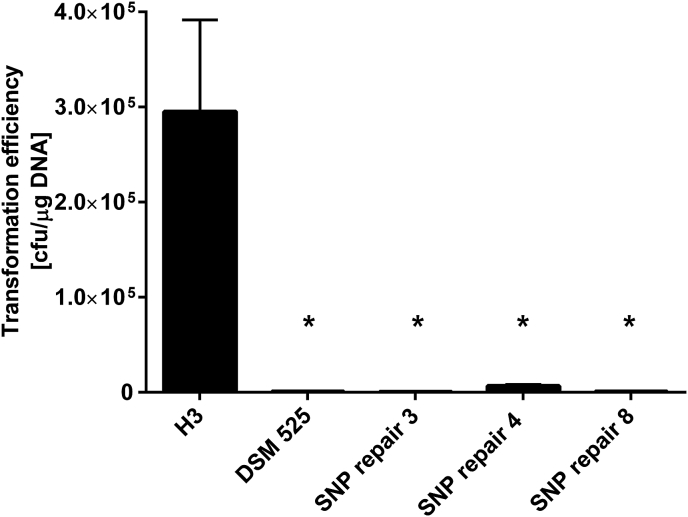
Histogram showing transformation efficiencies of *C. pasteurianum*-H3 SNP corrected mutants. Efficiencies are given of positive control *C. pasteurianum*-H3 (H3); negative control *C. pasteurianum* DSM 525 (DSM 525); the mutants in which the SNP in CLPA_c30550 is corrected back to wild type (DSM 525) genotype (SNP repair 3, 4, 8). Asterisks indicate statistical significance difference to H3 calculated by one-way ANOVA (*F*(5,18) = 7.716, *p* = 0.0005) with *a posteriori* Dunnett's multiple comparisons test.

It would be expected that restoration of the CLPA_c30550 SNP in strain 525-H3 to that of the wildtype DSM 525 strain would significantly reduce transformation frequencies. Accordingly, competent cells of three, randomly selected SNP repaired clones (3, 4 and 8) were prepared and their transformation efficiencies tested using *in vivo* methylated pMTL85151 plasmid DNA. As predicted the frequency of plasmid transfer obtained was reduced to that of the wildtype DSM 525 strain (Fig. 1).

Whilst our data unequivocally implicates CLPA_c30550 in DNA transfer, as in our previous study, the protein appears not to be a restriction enzyme. It does, however, exhibit some similarity with structural maintenance of chromosome (SMC) proteins. These play pivotal roles in chromosome dynamics in prokaryotes and eukaryotes. Members of the SMC protein family are involved in chromosome condensation, sister chromatid cohesion, chromosome partitioning, dosage compensation, DNA repair, DNA supercoiling and recombination [7]. In *B. subtilis* SMC proteins affect supercoiling *in vivo*, most likely by constraining positive supercoils, an activity which contributes to chromosome compaction and organization [8]. It is unclear how a point mutation of such an enzyme could lead to the observed increased transformation efficiency in *C. pasteurianum*.

Our study has highlighted the fact that barriers other than restriction modification can significantly affect DNA transfer rates in *C. pasteurianum*. Despite not being able to understand the basis of the improvement, the present study reinforces the utility of our strategy for isolating hypertransformable strains more suitable for genetic modification. Bacterial populations will always be composed of mutant-containing sub-populations and these will accumulate with time if they are neutral under the conditions maintained. Recent studies have for instance estimated the mutation rate in *E. coli* to be approx. 10^10^ per generation [9]. Despite the fact that a particular mutation will only be present in very low numbers, the method employed here (assuming the strain is poorly, or even, not transformable) is highly selective for those mutations that affect DNA transfer, as only cells that inherit the plasmid can survive on the selective media used. The method should prove applicable to clostridial species or even bacterium in general where low or non-existent gene transfer is an issue.

## Author contributions

AGH and HW undertook all the experimental work described in the study, with guidance from KMS, NPM and YZ. NPM and YZ conceived the study. AGH, NPM and YZ wrote the manuscript. All authors read and commented on the manuscript.

## Conflicts of interest

The authors declare no conflicts of interest.
