# Supplementary material for: Improving gene transfer in Clostridium pasteurianum through the isolation of rare hypertransformable variants
Source: Anaerobe. 2017 Dec;48:203–5. doi: 10.1016/j.anaerobe.2017.09.001 (PMC5734229; doi:10.1016/j.anaerobe.2017.09.001)
Supplement: Supplementary material [file mmc1.pdf]

SUPPLEMENTARY INFORMATION

Improving gene transfer in *Clostridium pasteurianum* through the isolation of rare hypertransformable variants

Alexander Grosse-Honebrink[a], Katrin M. Schwarz[a], Hengzheng Wang[a], Nigel P. Minton[a], Ying Zhang[a]*

[a]*Clostridia Research Group, BBSRC/EPSRC Synthetic Biology Research Centre (SBRC), University of Nottingham, University Park, Nottingham NG7 2RD, UK.*

*corresponding author: Ying.Zhang@nottingham.ac.uk

Electroporation of *Clostridium pasteurianum*

Electroporation of *C. pasteurianum* was done similar to Pyne et al. (2013). Briefly, 20 ml of 2x YTG broth were inoculated with cells from a well grown mid-exponential phase overnight culture (OD_{600} of 0.8 to 1.2) to a final OD_{600} of 0.05 and incubated for 3 to 4 h until an OD_{600} of 0.6 to 0.8 was reached. Cells were centrifuged (10 min, 8,500 x g, 4°C), re-suspended in 5 ml ice cold SMP buffer (270 mM sucrose, 1 mM $MgCl_2$, 5 mM sodium phosphate [pH 6.5]), centrifuged (10 min, 8,5000 x g, 4°C) another time and re-suspended in 600 µl ice cold SMP buffer. Of the 600-µl cell suspension 580 µl were transferred into a pre-chilled 0.4 cm electroporation cuvette (Sigma-Aldrich, Dorset, UK) containing 30 µl of 96% (v/v) pure non-denatured ethanol and 0.5 to 5 µg of methylated plasmid DNA. The cell-ethanol-plasmid suspension was incubated for 5 min on ice and, subsequently, electroporated using a Gene Pulser Xcell™ electroporation system (Bio-Rad, Hemel Hempstead, UK), a voltage of 1.8 kV, a capacitance of 25 µF and a resistance of ∞ Ω generating a time constant of 10–18 ms. Immediately after the pulse cells were transferred into pre-warmed 2x YTG broth supplemented with 40 µg/ml uracil, if required, and recovered for 6 to 16 h. Following, cells were centrifuged (10 min, 8,500 x g, RT), re-suspended in 250 µl PBS and plated on RCM agar plates containing the appropriate antibiotic selection (Table 1). Transformants typically took 16 to 24 h (replicative, non-integrative plasmids) or 48 h (allelic exchange plasmids) to appear. If transformation efficiencies were to be determined, 100 µl of the 250-µl re-suspension were serial diluted in a total of 1,000 µl PBS of which 100 µl were plated. Colonies were enumerated after a 24-h incubation. All centrifugation steps and washes were carried out anaerobically at 4°C or on ice. If needed, competent cells were frozen until further use. Therefore, cell pellets were re-suspended in 540 µl SMP buffer, supplemented with 60 µl DMSO (10% [v/v]) and frozen at -80 °C.

Construction of the Allelic Exchange (AE) KO plasmids

pMTL-AGH15: To generate an allelic exchange vector using *codA* (cytosine deaminase) as the heterologous counter (negative) selection marker (Cartman et al., 2012), the *codA* gene from pMTLSC7215 was fused with a promoterless *catP* by SOE (splicing by overlap extension) PCR as described previously (Ng et al., 2013) using the oligonucleotides codA_F_FseI,

codA_R, catP_F and catP_R_PmeI (Table S1), and cloned into pMTL-KS15 (Schwarz et al., 2017) via *Fse*I/*Pme*I sites. The resultant plasmid was designated pMTL-AGH15, the *codA* KO AE vector (Figure S1).

pMTL-AGH15-c30550: A 1127 bp fragment of the wild type allele was amplified flanking the SNP in 525-H3 by 599 bp down- and 527 bp upstream using primers Segr_LHA_SbfI_F and Segr_RHA_NheI_R (Table S1), then cloned into pMTL-AGH15 via *Sbf*I and *Nhe*I, yielding the SNP repair plasmid pMTL-AGH15-c30550.

Allelic Exchange (AE) KO procedure

For the restoration of the CLPA_c30550 SNP in strain 525-H3 by allelic exchange, pMTL-AGH15-c30550 were transformed into the strain 525-H3 and plated on RCM+Tm$_{15}$ agar. Following 48 h incubation, faster growing colonies were re-streaked twice onto RCM+Tm$_{15}$ agar plates and their identity as single crossover integrants confirmed by Sanger sequencing of the PCR amplified DNA fragment using appropriate primer pairs. Confirmed single crossover mutants were grown overnight in 5 ml RCM broth to allow the double crossover to occur, centrifuged (10 min, 8,500 x g, RT) and re-suspended in 250 µl PBS. A 100 µl aliquot of the suspended cells was serially diluted (up to 10$_{-7}$) in PBS and 100 µl of each dilution plated onto CBM agar plates containing 500 µg/ml fluorouracil (FC). After a 24 h incubation, 24 single, faster growing colonies were selected and re-streaked in the indicated order onto RCM+Tm$_{15}$, and RCM agar plates. Colonies, which lost the plasmid and either reverted back to the WT or carried the desired SNP restoration exhibited no growth on RCM+Tm^{15} agar plates but grew on RCM agar plates. These re-streaks were subjected to colony PCR, using gene specific primers that flanked the intended deletion, and the amplified DNA fragment subjected to Sanger sequencing to confirm the expected genotype.

Table S1: Primers used in this study. * Underlined: added restriction endonuclease recognition sites; Lower cases: 5’ overhang complementary sequences in SOE PCR.

Name	Sequence 5’ to 3’*
**Plasmid Specific Colony PCR Primers**	
traj_F	GCTTGGCAAGGTCATGATG
MCS_85_Rev	CGCTTATTCGCTTCGCTCAT
**Cloning Primers**	
codA_F_FseI	CAAGGCAAGACCGATCGGGCCGGCCGCAGG
codA_R	aattcaagttTCGTTCAACGTTTGTAATCGATGGCTTCTGGCTGC
catP_F	cgttgaacgaAACTTGAATTTGAGAGGGAACTTAG
catP_R_PmeI	AAAAAACAAATTTGAAGTTAACTATTTATCAATTCCTGC
Segr_LHA_SbfI_F	GAATTCCCTGCAGGTGCTTTGCTCTCTTATTCTCTCA
Segr_RHA_NheI_R	AATTCGCTAGCAAAGGAATGTAGCAGTGCGA
**Sequencing Primers**	
Segr_flank_F	TCCAATTCCAACAACTCCATCA
Segr_flank_R	GGAAGGCCTAAGTGCAATACA

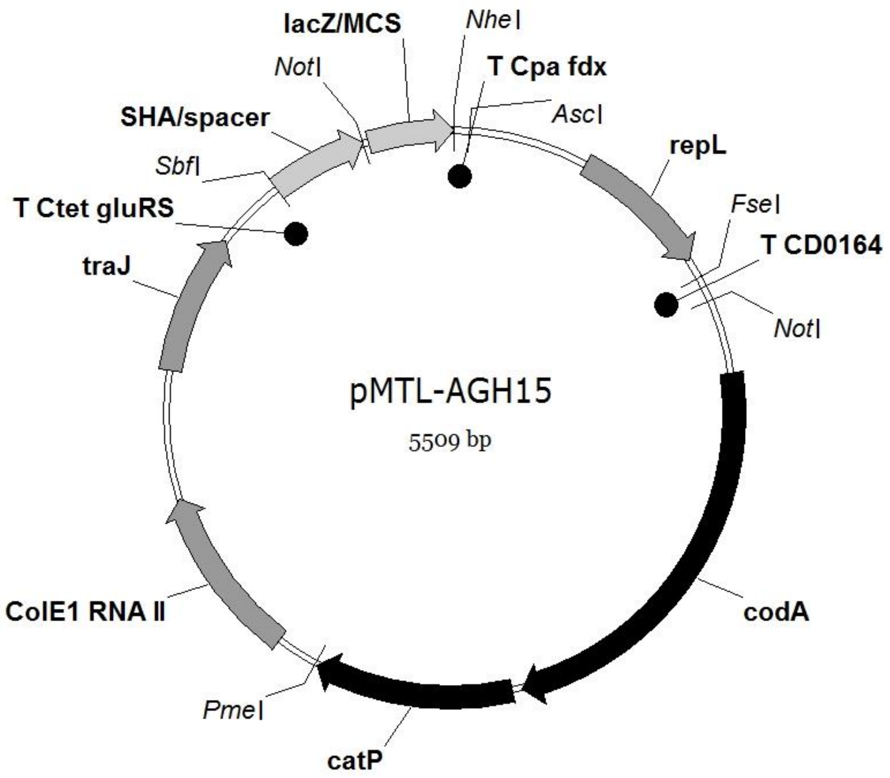

Figure S1. Plasmid map of pMTL-AGH15. (B). Key: T CD0164, a transcriptional terminator isolated from downstream of the *C. difficile* strain 630 CD0164 gene; *codA*, a *E. coli*-derived gene encoding cytosine deaminase; *catP*, a *C. perfringens*-derived gene encoding chloramphenicol acetyltransferase; ColE1, the replication origin of plasmid ColE1; *traJ*, transfer function of the RP4 *oriT* region; T Ctet *gluRS*, transcriptional terminator of *C. tetani* E88 glutamyl-tRNA synthetase; T Cpa *fdx*, transcriptional terminator of the ferredoxin gene of *C. pasteurianum*; repL, replication region of the *Bacillus subtilis* plasmid pIM13.

References

Pyne, M.E., Moo-Young, M., Chung, D.A. and Chou, C.P., 2013. Development of an electrotransformation protocol for genetic manipulation of *Clostridium pasteurianum*. *Biotechnology for biofuels*, 6(1), p.50. doi: 10.1186/1754-6834-6-50

Cartman, S.T., Kelly, M.L., Heeg, D., Heap, J.T. and Minton, N.P., 2012. Precise manipulation of the *Clostridium difficile* chromosome reveals a lack of association between the *tcdC* genotype and toxin production. *Applied and environmental microbiology*, 78(13), pp.4683-4690. doi: 10.1128/AEM.00249-12

Ng, Y.K., Ehsaan, M., Philip, S., Collery, M.M., Janoir, C., Collignon, A., Cartman, S.T. and Minton, N.P., 2013. Expanding the repertoire of gene tools for precise manipulation of the *Clostridium difficile* genome: allelic exchange using pyrE alleles. *PLoS One*, 8(2), p.e56051. doi: 10.1371/journal.pone.0056051

Schwarz, K.M., Grosse-Honebrink, A., Derecka, K., Rotta, C., Zhang, Y. and Minton, N.P., 2017. Towards improved butanol production through targeted genetic modification of *Clostridium pasteurianum*. *Metabolic engineering*, 40, pp.124-137. doi: 10.1016/j.ymben.2017.01.009
